# External Validation of Six Liver Functional Reserve Models to predict Posthepatectomy Liver Failure after Major Resection for Hepatocellular Carcinoma

**DOI:** 10.7150/jca.58726

**Published:** 2021-06-26

**Authors:** Guangmeng Guo, Zhengqing Lei, Xuewu Tang, Weihu Ma, Anfeng Si, Pinghua Yang, Qi Li, Zhimin Geng, Jiahua Zhou, Zhangjun Cheng

**Affiliations:** 1 Hepato-pancreato-biliary center, Zhongda Hospital, School of Medicine, Southeast University, Nanjing, China.; 2 Department of Surgical Oncology, Qin Huai Medical District of Eastern Theater General Hospital, Nanjing, China.; 3 Department of Minimally Invasive Surgery, the Eastern Hepatobiliary Surgery Hospital, Second Military Medical University, Shanghai, China.; 4 Department of Hepatobiliary Surgery, the First Affiliated Hospital of Xi'an Jiaotong University, Xi'an, China.

**Keywords:** hepatocellular carcinoma, major hepatectomy, preoperative prediction, posthepatectomy liver failure

## Abstract

**Objective:** To validate and compare the predictive ability of albumin-bilirubin model (ALBI) with other 5 liver functional reserve models (APRI, FIB4, MELD, PALBI, King's score) for posthepatectomy liver failure (PHLF) in patients with hepatocellular carcinoma (HCC) who underwent major hepatectomy.

**Methods:** Data of patients undergoing major hepatectomy for HCC from 4 hospitals between January 01, 2008 and December 31, 2019 were retrospectively analyzed. PHLF was evaluated according to the definition of the 50-50 criteria. Performances of six liver functional reserve models were determined by the area under the receiver operating characteristic curve (AUC), calibration plot and decision curve analysis.

**Results:** A total of 745 patients with 103 (13.8%) experienced PHLF were finally included in this study. Among six liver functional reserve models, ALBI showed the highest AUC (0.64, 95% CI: 0.58-0.69) for PHLF. All models showed good calibration and greater net benefit than treating all patients at a limit range of threshold probabilities, but the ALBI demonstrated net benefit across the largest range of threshold probabilities. Subgroup analysis also showed ALBI had good predictive performance in cirrhotic (AUC=0.63) or non-cirrhotic (AUC=0.62) patients.

**Conclusion:** Among the six models, the ALBI model shows more accurate predictive ability for PHLF in HCC patients undergoing major hepatectomy, regardless of having cirrhosis or not.

## Introduction

Hepatocellular carcinoma (HCC) is the fifth most common malignancy in the world. Partial hepatectomy (PH) is still the mainstay of curative-intent treatment for HCC 1. With improvements in surgical techniques and perioperative management, increasing number of HCC patients are able to undergo PH. However, postoperative morbidity and mortality still exists, and posthepatectomy liver failure (PHLF) remains the most common cause of mortality particularly in patients who underwent major hepatectomy 2. The reported incidence of PHLF is 0.7%-9.1%, especially in patients underwent major hepatectomy as high as 58.22%, and PHLF is shown as the major cause (between 18% and 75%) of postoperative mortality 3-4. In consideration of such a high incidence, accurate prediction of PHLF is very important for patient selection and perioperative management in HCC patients following major hepatectomy.

Previously, some liver functional reserve models have been created to assess liver function and predict posthepatectomy outcomes in patients with HCC. MELD model was used to predict survival after hepatectomy of HCC patients 5-7, APRI, FIB4 and King's score models were created to assess liver fibrosis and cirrhosis of HBV and HCV patients 8-12. These models were gradually adopted to predict PHLF and exhibited a certain predictive ability 13-16. Recently, ALBI and PALBI model were developed to evaluate liver function and used to predict PHLF after hepatectomy for HCC 17-23. Although various studies demonstrated the usefulness of these liver functional reserve models in prediction of PHLF, few studies compared their accuracy in HCC patients undergoing major hepatectomy.

The aim of this study is to validate and compare the predictive ability of the ALBI model with other 5 existing liver functional reserve models (PALBI, APRI, MELD, FIB4, and King's score) for PHLF in patients with hepatocellular carcinoma after major hepatectomy.

## Patients and methods

### Study design

A multicentric retrospective analysis of patients with hepatocellular carcinoma who underwent major hepatectomy (≥ 3 segments) from the Eastern Hepatobiliary Surgery Hospital in Shanghai, Zhongda Hospital, Southeast university in Nanjing, Qin Huai Medical District of Eastern Theater General Hospital in Nanjing and The First Affiliated Hospital of Xi'an Jiao Tong University in Xi'an between January 01, 2008 and December 31, 2019 were recorded. Inclusion criteria are obeyed strictly as follows: (1) pathologically confirmed as hepatocellular carcinoma; (2) underwent major hepatectomy, major hepatectomy is defined as resection of Couinaud's segmentation 3 and above 4; (3) no anti-tumor treatment before surgery, such as ablation, TACE; (4) no major blood vessel invasion, bile duct cancer thrombus and distant metastasis; (5) age ≥18. Exclusion criteria: (1) intraoperative radiofrequency ablation, particle placement and other treatments; (2) data were missing on the fifth day after surgery, and PHLF could not be defined.

### Surgical technique

The technique of hepatectomy has been previously described 24. Parenchymal transection was usually achieved under intermittent pedicle clamping (15-minute occlusion and 5-minute reperfusion). Under anesthesia, the hemodynamic management aimed to maintain low central venous pressure to minimize blood loss with reduced volume perfusion.

### Variables of interest

Data were collected by direct extraction from electronic health records, complemented by manual curation. Variables of interest in the dataset included: demographics (age, gender, BMI), laboratory measurements (TBIL, ALB, PLT, PT, INR, ALT, AST and Cr), underlying liver disease (HBsAg, HBeAg, HBV-DNA, anti-HCV and antiviral therapy), radiology reports (ascites and cirrhosis), surgical factors (clamping time, blood loss and intraoperative transfusion) and pathological reports (tumor size, tumor number, microvascular invasion and tumor differentiation).

### Liver functional reserve models

A total of 6 liver functional reserve models were investigated: ALBI, PALBI, MELD, APRI, FIB4, King's score which were depicted in detail in Table S1. Comparison of these models was performed through discrimination.

### Outcomes and definitions

The main outcome of this study was PHLF. The “50-50 criteria” was used as the definition of PHLF in this study: prothrombin activity < 50% and posthepatectomy serum bilirubin > 50 μmol/L, fifth day after operation 25. To provide a better overview of the predictive abilities on distinct patient populations, this study performed subgroup analyses based on liver cirrhosis according to imaging findings.

### Statistical analysis

Continuous variables were expressed as median with the interquartile range (IQR). Categorical variables were presented as numbers and percentages. The X^2^ test or Fisher's exact test was used to analyze categorical variables and the Mann-Whitney ranked sum test for continuous variables. The discrimination ability of models was evaluated by the Receiver Operating Characteristic Curve (ROC) and area under the curve (AUC). Comparisons between the ROC curves were performed using the corresponding DeLong's tests. We assessed calibration by visualizing calibration of predicted vs. observed risk using loess-smoothed plots. Decision curve analyses were done using the rmda package in R 26. A two-tailed P values <0.05 was considered statistically significant. Statistical analysis was performed using Stata/SE 15.1 (College Station, TX77845, USA) and R (Version 4.0.2, https://www.r-project.org).

## Results

### Baseline characteristics of study population

**Figure 1:**
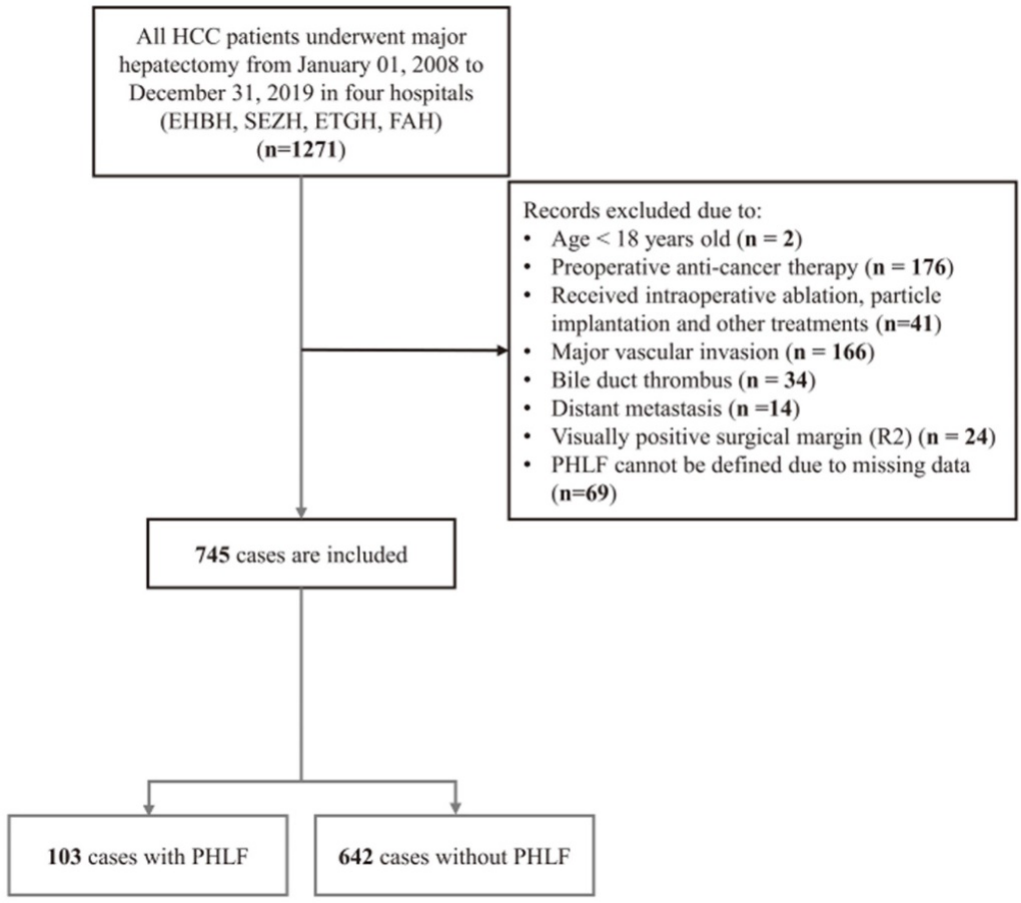
Flow chart of the study.

As was shown in Figure 1, a total of 745 HCC patients were enrolled in the present study, comprising 624 (83.8%) men, with a median age of 53 years (IQR, 45-60 years). Among them, 103 patients experienced PHLF, accounting for 13.8%. The median value of ALBI, PALBI, APRI, MELD, FIB4 and King's score is -2.8 (-3.00 to -2.56), -2.56 (-2.73 to -2.37), 0.52 (0.30-0.88), 7 (6-7), 1.79 (1.06-2.83), and 11.0 (6.06-18.5), respectively. According to the ALBI grade, the majority of patients had grade 1 (532/745, 71.4%), 28.5% (212/745) as grade 2 and 0.1% (1/745) as grade 3. The patient's basic characteristics, preoperative clinical indicators and six model information are shown in Table 1. There was statistical significance difference in terms of BMI, HBV-DNA, Cirrhosis, TBIL, ALB, PLT, PT/INR, ALT, blood loss, intraoperative transfusion, and the six liver functional reserve models.

### Significant univariable predictors for PHLF

Univariate logistic regression was performed on the aforementioned possible clinical risk factors (Table 2). The possible discriminating univariable factors for PHLF were BMI, HBV-DNA, TBIL, ALB, PLT, prothrombin time, INR, blood loss, while the most accurate one was prothrombin time (AUC: 0.64, 95% CI: 0.58-0.69).

### Evaluation of liver functional reserve models for PHLF

**Figure 2:**
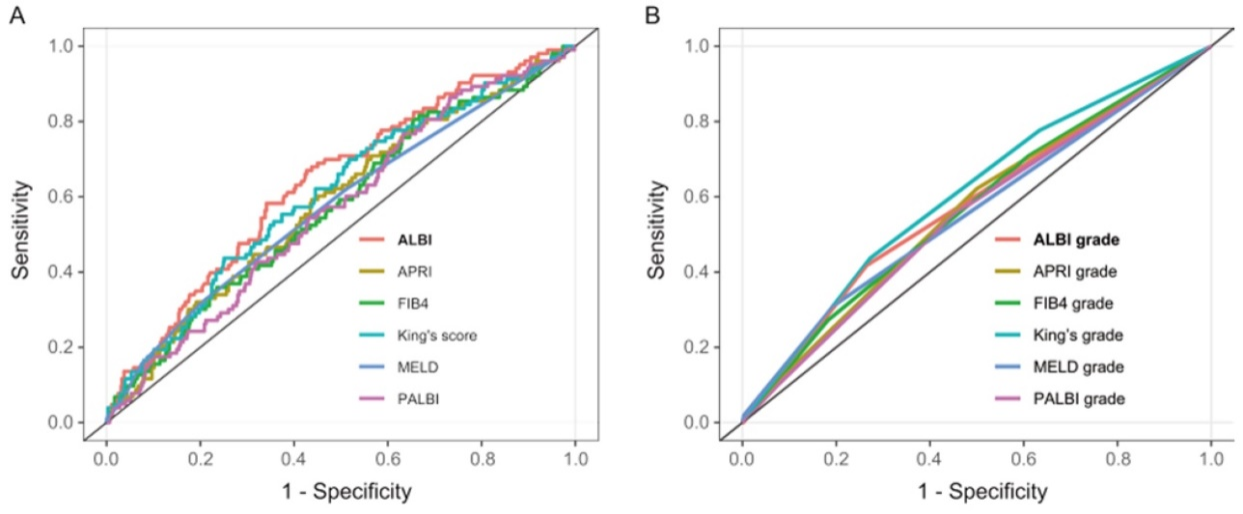
Receiver operating characteristic (ROC) curves of six liver functional reserve models (A) and hierarchical models (B) predicting posthepatectomy liver failure (PHLF) for hepatocellular carcinoma (HCC) patients who underwent major hepatectomy.

**Figure 3:**
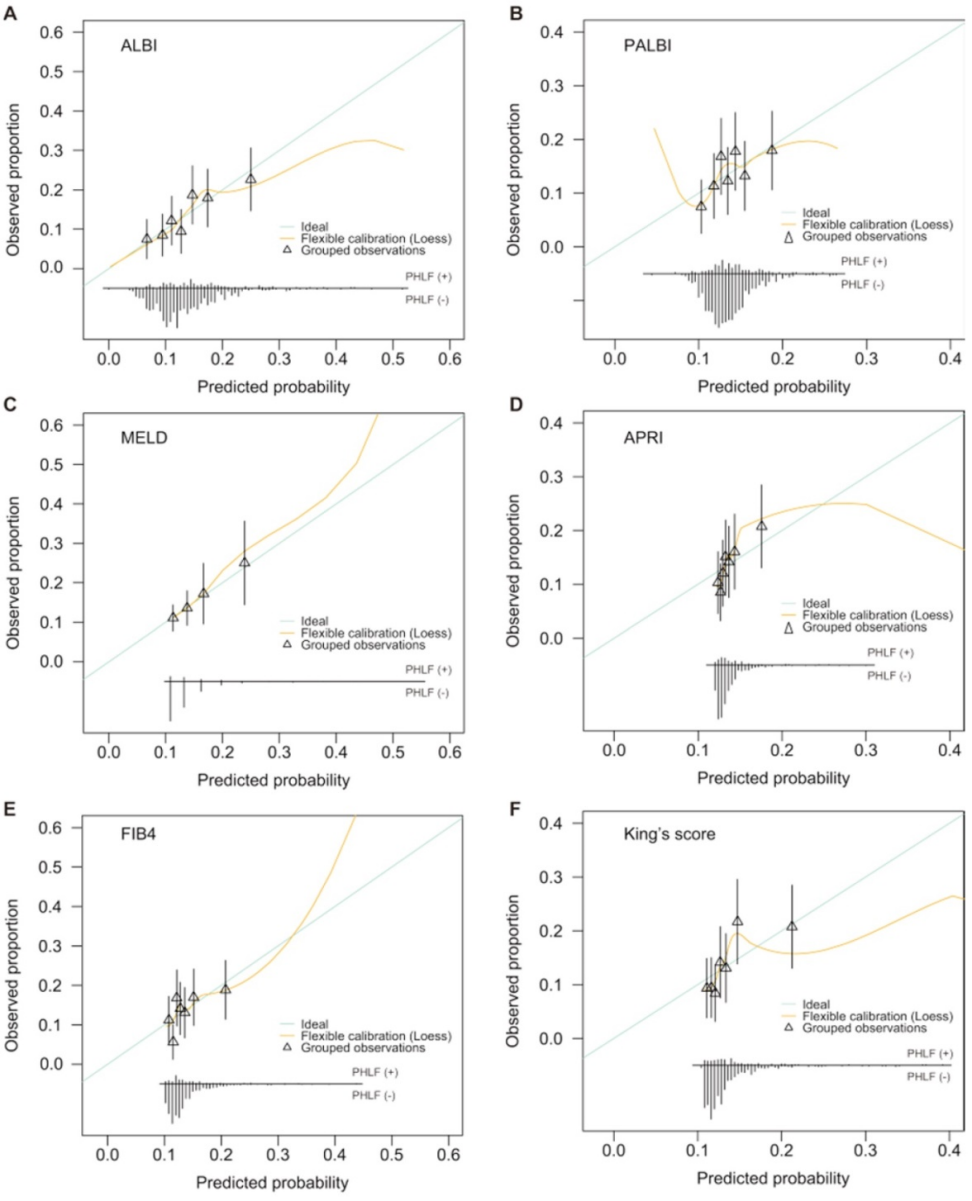
Calibration plots for six models estimating PHLF probabilities. A: ALBI; B: PALBI; C: MELD; D: APRI; E: FIB4; F: King's score.

AUCs for the six models were as follows: ALBI AUC 0.64 (95% CI: 0.58-0.69), MELD AUC 0.58 (95% CI: 0.52-0.64), APRI AUC 0.59 (95% CI: 0.53-0.64), FIB4 AUC 0.57 (95% CI: 0.51-0.63), PALBI AUC 0.57 (95% CI: 0.51-0.63), and King's score AUC 0.61 (95% CI: 0.55-0.67) (Table 3 and Figure 2A). ALBI model had the greatest AUC for predicting PHLF. Furthermore, when the six models were categorized into 3 classifications, most of their prediction performance will declined with exception of King's score (Table 3 and Figure 2B). For all models, calibration appeared visually good (Figure 3).

### Decision curve analyses to assess clinical utility

**Figure 4:**
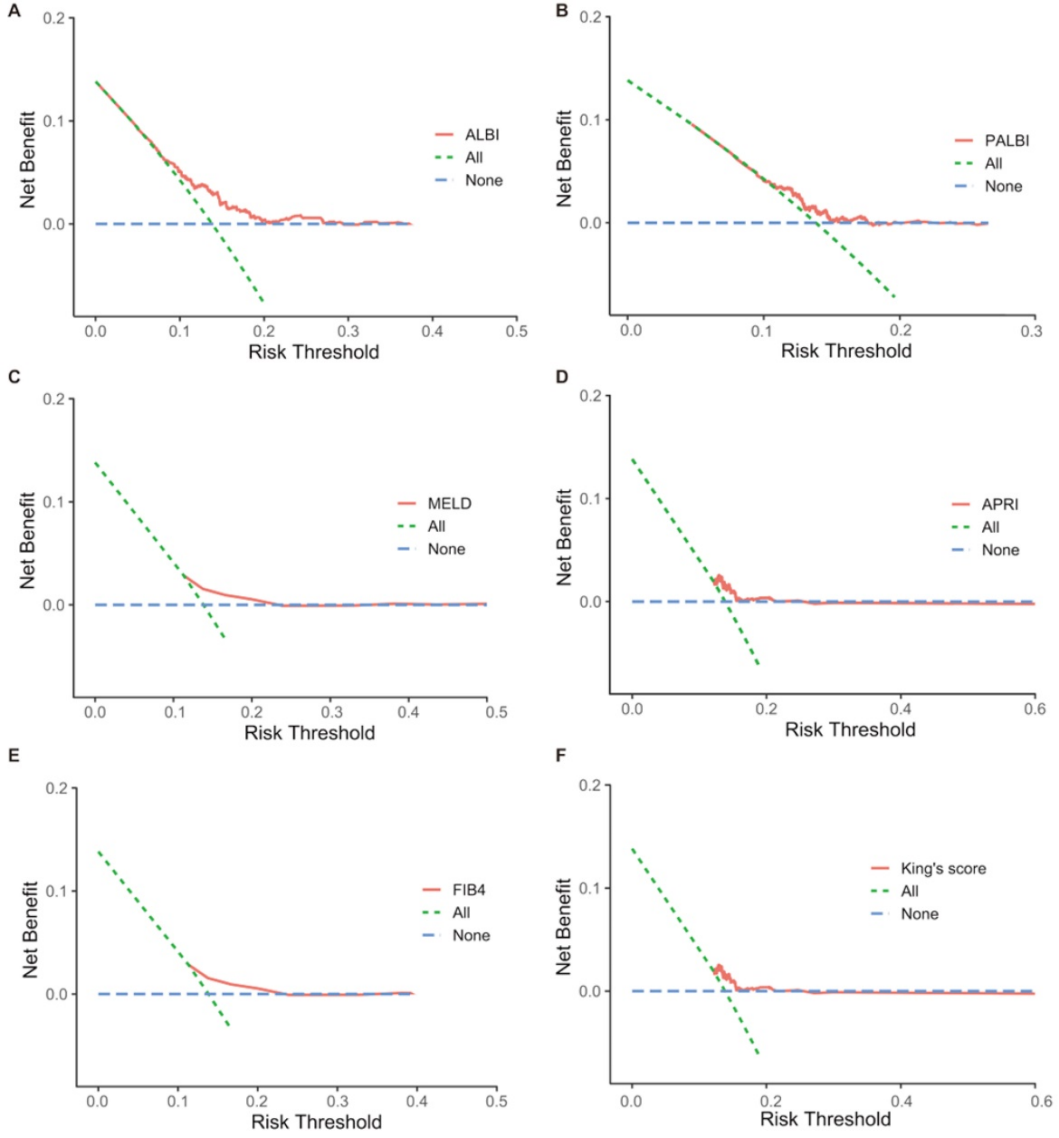
Decision curve analysis comparing net benefit of each liver functional reserve model for PHLF. A: ALBI; B: PALBI; C: MELD; D: APRI; E: FIB4; F: King's score.

This study compared net benefit for each model to the strategies of treating all patients, treating no patients for PHLF. Although all models showed greater net benefit than treating all patients at a limit range of threshold probabilities, the ALBI demonstrated net benefit across the largest range of threshold probabilities (Figure 4).

### Subgroup analysis

Finally, subpopulation analyses were done in the patients with or without cirrhosis to assess the overall performance of ALBI compared with the other 5 models. Subgroup analysis was presented in a forest plot (Figure S1). In cirrhotic patients, ALBI and PALBI had the better predictive accuracy (AUC = 0.63 and 0.60, respectively) compared with other 4 models. In subgroup patients without cirrhosis, ALBI, MELD, FIB4 and King's score had significant predictive accuracy (P = 0.012, 0.030, 0.011, 0.007 and AUC = 0.62, 0.59, 0.61 and 0.63, respectively).

## Discussion

Liver failure is one of the leading causes of perioperative death in patients with hepatocellular carcinoma after major hepatectomy. This study applied the “50-50 criteria” proposed by Silvio Balzan et al. in 2005 as a diagnostic criterion for liver failure. In our study, the incidence of liver failure after major liver resection is 13.8%, the 90-day mortality of patients with PHLF was significantly higher than the patients who did not experience liver failure (38.6% vs. 26.2%, P = 0.011) in our multi-institutional cohort. Selection criteria for major hepatectomy and prediction of the individual PHLF risk are debatable. Accurate assessment of preoperative liver reserve function is one of the keys to reducing the occurrence and death of liver failure after major liver resection 27. Clinicians have been preferring to use new classification of objective indicators to assess liver function more accurately. Therefore, MELD, APRI, FIB4, King's score, ALBI and PALBI models have been proposed successively. This study aimed at evaluating these six common models of PHLF in HCC patients undergoing major hepatectomy.

In this article, 745 patients with hepatocellular carcinoma who underwent major hepatectomy were retrospectively analyzed. As for univariable risk factor, BMI, HBV-DNA, TBIL, ALB, PLT, prothrombin time, INR, and blood loss were predictors for PHLF. Thus, the factors which were related to patients' demographic, HBV-related, liver related and surgical factors have been shown the associations with PHLF. The result was partially consistent with Kauffmann et al. and Shoup et al. who validate TBIL and coagulation as risk factors of PHLF 28-29.

As an integrated variable, ALBI indicated the best predictive accuracy, regardless of having cirrhosis or not. Although these models are calculated using objective indicators, the numerical results and the accuracy of predictions vary a lot. The ALBI model proposed in 2015 was simply calculated from two indicators, ALB and TBIL, and many literatures have confirmed its accuracy in assessing liver function in patients with hepatocellular carcinoma 30-31. At the same time, ALBI and King's score models were equally statistically significant in predicting PHLF after major liver resection (P = 0.395). As verified by this article, for HCC patients undergoing major hepatectomy, the ALBI model has a better ability to predict PHLF then PALBI (P = 0.005) and MELD (P = 0.007). This result was in accordance with the study reported by Alexander M. that ALBI can predict PHLF better than MELD after hepatectomy 21. Subgroup analysis was then performed based on patients with or without cirrhosis. For cirrhotic patients, except ALBI, PALBI may be also a good choice for predicting PHLF. Moreover, for patients without cirrhosis, ALBI, MELD, FIB4 and King's score may be more suitable.

The strengths of this study lie in the large patient population. However, the present study has some limitations. First, it is a retrospective study with inherent shortcoming. Second, although our data are from four well-known hospitals, they are all from Chinese people, whether our conclusion is suitable in patients from the West remains uncertain. Third, there is no data on remnant liver volume and ICG-15, which may be useful in predicting PHLF. Further external validation of our results is needed.

## Conclusion

In conclusion, among the six liver functional reserve models (ALBI, APRI, FIB4, MELD, PALBI, King's score), the ALBI exhibits more accurate predictive ability for PHLF in HCC patients undergoing major hepatectomy, regardless of having cirrhosis or not.

## Supplementary Material

Supplementary figure and table.

## Figures and Tables

**Table 1:** Baseline characteristics of patients

Variable	Total (n = 745)	Without PHLF (n = 642)	PHLF (n = 103)	*P* value
**Demographics**				
Age, years	53.0 (45.0-60.0)	53.0 (44.0-60.0)	54.0 (47.5-60.5)	0.154
**Sex**				0.724
Female	121 (16.2%)	106 (16.5%)	15 (14.6%)	
Male	624 (83.8%)	536 (83.5%)	88 (85.4%)	
BMI, Kg/m^2^	23.3 (21.5-24.8)	23.3 (21.5-24.9)	22.5 (21.2-24.2)	0.038
**Diabetes**				0.060
No	694 (93.2%)	601 (93.6%)	93 (90.3%)	
Yes	51 (6.8%)	41 (6.4%)	10 (9.7%)	
**Underlying liver disease**				
HBsAg				0.492
Negative	165 (22.1%)	139 (21.7%)	26 (25.2%)	
Positive	580 (77.9%)	503 (78.3%)	77 (74.8%)	
**HBeAg**				0.511
Negative	600 (80.5%)	520 (81.0%)	80 (77.7%)	
Positive	145 (19.5%)	122 (19.0%)	23 (22.3%)	
HBV-DNA, Log10 IU/mL	0.00 (0.00-4.53)	0.00 (0.00-4.47)	3.00 (0.00-5.00)	0.022
**Anti-HCV**				1.000
No	736 (98.8%)	634 (98.8%)	102 (99.0%)	
Yes	9 (1.2%)	8 (1.2%)	1 (1.0%)	
**Antiviral therapy**				0.280
No	604 (81.1%)	523 (81.4%)	81 (78.6%)	
Yes	129 (17.3%)	107 (16.7%)	22 (21.4%)	
Data Missing	12 (1.6%)	12 (1.9%)	0 (0.0%)	
**Radiological findings**				
Ascites				0.372
No	669 (89.8%)	579 (90.2%)	90 (87.4%)	
Mild	76 (10.2%)	63 (9.8%)	13 (12.6%)	
**Cirrhosis**				0.033
No	437 (58.7%)	387 (60.3%)	50 (48.5%)	
Yes	308 (41.3%)	255 (39.7%)	53 (51.5%)	
**Laboratory measurements**				
TBIL, μmol/L	13.4 (10.0-18.0)	13.2 (10.0-17.6)	15.0 (10.5-19.8)	0.029
ALB, g/L	41.5 (39.0-44.0)	42.0 (39.0-44.3)	40.0 (38.0-42.5)	<0.001
PLT, ×10^9^/L	182 (138-237)	184 (141-240)	164 (120-223)	0.011
PT, seconds	12.0 (11.3-12.9)	12.0 (11.2-12.7)	12.4 (11.9-13.4)	<0.001
INR	1.00 (0.98-1.03)	1.00 (0.98-1.03)	1.00 (1.00-1.09)	0.001
ALT, U/L	36.0 (24.0-52.4)	35.0 (23.0-52.0)	41.9 (26.3-65.0)	0.012
AST, U/L	36.0 (23.0-58.0)	35.0 (23.0-56.0)	40.0 (25.0-63.0)	0.069
Cr, μmol/L	68.0 (59.0-77.0)	68.0 (59.0-76.8)	67.0 (58.0-77.5)	0.848
**Surgical factor**				
Clamping time, min	16.0 (9.00-22.0)	16.0 (9.00-22.0)	16.0 (10.0-20.0)	0.411
Blood loss, mL	400 (200-800)	350 (200-700)	500 (200-1000)	0.003
**Intraoperative transfusion**				0.005
No	519 (69.7%)	460 (71.7%)	59 (57.3%)	
Yes	226 (30.3%)	182 (28.3%)	44 (42.7%)	
**Tumor factor**				
Tumor size, cm	9.00 (5.85-12.7)	9.00 (5.20-12.4)	10.0 (7.00-13.0)	0.064
**Tumor number**				0.487
1	626 (84.0%)	543 (84.6%)	83 (80.6%)	
2	118 (15.9%)	98 (15.3%)	20 (19.4%)	
3	1 (0.1%)	1 (0.1%)	0 (0.0%)	
**Microvascular invasion**				0.280
No	289 (38.8%)	254 (39.6%)	35 (44.0%)	
Yes	456 (61.2%)	388 (60.4%)	68 (66.0%)	
**Differentiation**				0.173
well	7 (0.9%)	7 (1.1%)	0 (0.0%)	
moderate	718 (96.4%)	621 (96.7%)	97 (94.2%)	
poor	20 (2.7%)	14 (2.2%)	6 (5.8%)	
**Liver functional reserve models**				
ALBI	-2.80 (-3.00 - -2.56)	-2.81 (-3.01 - -2.58)	-2.66 (-2.88 - -2.44)	<0.001
**ALBI grade**				0.004
1	532 (71.4%)	472 (73.5%)	60 (58.3%)	
2	212 (28.5%)	169 (26.3%)	43 (41.7%)	
3	1 (0.1%)	1 (0.2%)	0 (0.0%)	
MELD	7.00 (6.00-7.00)	7.00 (6.00-7.00)	7.00 (6.00-8.00)	0.007
**MELD grade**				0.007
1	588 (78.9%)	517 (80.5%)	71 (68.9%)	
2	153 (20.5%)	123 (19.2%)	30 (29.1%)	
3	4 (0.6%)	2 (0.3%)	2 (2.0%)	
PALBI	-2.56 (-2.73 - -2.37)	-2.56 (-2.74 - -2.38)	-2.50 (-2.65 - -2.33)	0.026
**PALBI grade**				0.153
1	398 (53.4%)	352 (54.8%)	46 (44.7%)	
2	290 (38.9%)	243 (37.9%)	47 (45.6%)	
3	57 (7.7%)	47 (7.3%)	10 (9.7%)	
APRI	0.52 (0.30-0.88)	0.50 (0.29-0.85)	0.62 (0.38-1.05)	0.006
**APRI grade**				0.063
1	360 (48.3%)	321 (50.0%)	39 (37.9%)	
2	321 (43.1%)	269 (41.9%)	52 (50.5%)	
3	64 (8.6%)	52 (8.1%)	12 (11.6%)	
FIB4	1.79 (1.06-2.83)	1.74 (1.04-2.77)	2.04 (1.38-3.37)	0.015
**FIB4 grade**				0.051
1	280 (37.6%)	250 (38.9%)	30 (29.1%)	
2	320 (43.0%)	275 (42.8%)	45 (43.7%)	
3	145 (19.4%)	117 (18.3%)	28 (27.2%)	
King's score	11.0 (6.06-18.5)	10.4 (5.88-17.8)	14.0 (8.60-23.0)	<0.001
**King's grade**				0.001
1	258 (34.6%)	235 (36.6%)	23 (22.3%)	
2	267 (35.8%)	232 (36.1%)	35 (34.0%)	
3	220 (29.5%)	175 (27.3%)	45 (43.7%)	

**Abbreviations:** PHLF, post-hepatectomy liver failure; HCC, hepatocellular carcinoma; BMI, body mass index; HBsAg, Hepatitis B surface antigen; HBeAg, Hepatitis B e-antigen; HBV-DNA, hepatitis B virus deoxyribonucleic acid; Anti-HCV, hepatitis C virus antibody; TBIL, total bilirubin; ALB, albumin; PLT, platelet; PT, prothrombin time; INR, international normalized ratio; ALT, alanine aminotransferase; AST, aspartate aminotransferase; Cr, creatinine; OR, odds ratio; CI, confidence interval; ALBI, albumin-bilirubin; PALBI, platelet-albumin-bilirubin; APRI, AST to platelet ratio index; MELD, model for end-stage liver disease; FIB4, fibrosis index based on 4 factors.

**Table 2:** AUCs of univariable predictors for PHLF in HCC patients undergoing major hepatectomy

Predictor	AUC	95%CI	*P* value
**Demographics**			
Age, years	0.54	0.48-0.61	0.154
Sex (Male/Female)	0.51	0.45-0.57	0.751
BMI, Kg/m^2^	0.44	0.38-0.50	0.039
Antiviral therapy (Yes/No)	0.52	0.46-0.58	0.476
**Underlying liver disease**			
HBsAg (Positive/Negative)	0.48	0.42-0.54	0.558
HBeAg (Positive/Negative)	0.52	0.46-0.58	0.587
Anti-HCV (Positive/Negative)	0.50	0.44-0.56	0.964
HBV-DNA, U/mL	0.58	0.52-0.64	0.016
**Radiological findings**			
Cirrhosis (Yes/No)	0.56	0.50-0.62	0.056
Ascites (Yes/No)	0.51	0.45-0.56	0.875
**Laboratory measurements**			
TBIL, μmol/L	0.57	0.51-0.62	0.029
ALB, g/L	0.39	0.33-0.45	<0.001
PLT, ×10^9^/L	0.42	0.36-0.48	0.011
PT, seconds	0.64	0.58-0.69	<0.001
INR	0.60	0.55-0.66	<0.001
ALT, U/L	0.58	0.52-0.64	0.012
AST, U/L	0.56	0.50-0.62	0.069
Cr, μmol/L	0.51	0.45-0.56	0.848
**Surgical factor**			
Blood loss, ml	0.59	0.53-0.65	0.004
Intraoperative transfusion (Yes/No)	0.57	0.51-0.63	0.019
Clamping time, min	0.47	0.41-0.54	0.413
**Tumor factor**			
Tumor size, cm	0.56	0.49-0.62	0.064
Tumor number	0.52	0.46-0.58	0.520
Microvascular invasion	0.53	0.48-0.58	0.281
Differentiation	0.53	0.49-0.57	0.173

**Abbreviations:** AUC, area under the curve; PHLF, post-hepatectomy liver failure; HCC, hepatocellular carcinoma; CI, confidence interval; BMI, body mass index; HBsAg, Hepatitis B surface antigen; HBeAg, Hepatitis B e-antigen; HBV-DNA, hepatitis B virus deoxyribonucleic acid; Anti-HCV, hepatitis C virus antibody; TBIL, total bilirubin; ALB, albumin; PLT, platelet; PT, prothrombin time; INR, international normalized ratio; ALT, alanine aminotransferase; AST, aspartate aminotransferase; Cr, creatinine.

**Table 3:** Discriminative ability of each liver functional reserve model in cohort

Model	AUC	95% CI	*P* value	DeLong's test for two correlated ROC curves
ALBI	0.64	0.58-0.69	<0.001	Ref.
ALBI Grade 1/2/3 (<-2.6/-2.6 ≤ -1.39 /> -1.39)	0.58	0.52-0.63	0.013	<0.001
PALBI	0.57	0.51-0.63	0.026	0.005
PALBI Grade 1/2/3 (≤ -2.53, -2.53-2.09, > -2.09)	0.55	0.50-0.61	0.09	0.001
APRI	0.59	0.53-0.64	0.006	0.153
APRI Grade, 1/2/3 (<0.5/0.5-1.5/>1.5)	0.56	0.51-0.62	0.035	0.039
MELD	0.58	0.52-0.64	0.012	0.007
MELD Grade, 1/2/3 (<8/8-12/>12)	0.56	0.51-0.61	0.053	0.012
FIB4	0.57	0.51-0.63	0.015	0.094
FIB4 Grade 1/2/3 (<1.45/1.45-3.25/>3.25)	0.57	0.51-0.62	0.028	0.062
King's score	0.61	0.55-0.67	<0.001	0.395
King's grade 1/2/3 (<7.6/7.6-16.7/16.7)	0.60	0.55-0.66	<0.001	0.351

**Abbreviations**: AUC, area under the curve; CI, confidence interval; ROC, receiver operating characteristic curve; ALBI, albumin-bilirubin; PALBI, platelet-albumin-bilirubin; APRI, AST to platelet ratio index; MELD, model for end-stage liver disease; FIB4, fibrosis index based on 4 factors.

## Abbreviations

PHLF: post-hepatectomy liver failure
HCC: hepatocellular carcinoma
CTP: Child-Turcotte-Pugh classification
ALBI: albumin-bilirubin
PALBI: platelet-albumin-bilirubin
APRI: AST to platelet ratio index
MELD: model for end-stage liver disease
FIB4: fibrosis index based on 4 factors
HCV: hepatitis C virus
HBV: hepatitis B virus
BMI: body mass index
HBsAg: Hepatitis B surface antigen
HBeAg: Hepatitis B e-antigen
HBV-DNA: hepatitis B virus deoxyribonucleic acid
Anti-HCV: hepatitis C virus antibody
TBIL: total bilirubin
ALB: albumin
PLT: platelet
PT: prothrombin time
INR: international normalized ratio
ALT: alanine aminotransferase
AST: aspartate aminotransferase
Cr: creatinine
OR: odds ratio
CI: confidence interval
ROC: Receiver Operating Characteristic Curve
AUC: area under the curve
IQR: interquartile range
