# Supplementary material for: External Validation of Six Liver Functional Reserve Models to predict Posthepatectomy Liver Failure after Major Resection for Hepatocellular Carcinoma
Source: J Cancer. 2021 Jun 26;12(17):5260–7. doi: 10.7150/jca.58726 (PMC8317527; doi:10.7150/jca.58726)
Supplement: Supplementary file 1 — Supplementary figure and table. [file jcav12p5260s1.pdf]

Table S1. Formula of 6 liver functional reserve models.

Liver Functional Reserve Model	Formula
**ALBI**	Log (Bilirubin[μmol/L]) × 0.66 + Albumin[g/L] × (−0.085)
**MELD**	10 × ((0.957 × ln (Creatinine[μmoI/L])) + (0.378 × ln (Bilirubin[μmol/L])) + (1.12 × ln (INR))) + 6.43
**APRI**	((AST[U/L] / upper limit of normal) / Platelet[10^9/L]) × 100
**FIB4**	(Age[years] × AST[U/L]) / (Platelet[10^9/L] × ALT[U/L]$^{1/2}$)
**PALBI**	(2.02 × log10 Bilirubin[μmol/L]) – (0.37 × (log10 Bilirubin[μmol/L])^2) − 0.04 × Albumin[g/L] − 3.48 × log10 Platelets[10^9/L] + 1.01 × (log10 Platelets[10^9/L])^2
**King's score**	Age[years] × AST[U/L] × INR/(Platelets[10^9/L])

Abbreviations: ALBI, albumin-bilirubin; MELD, model for end-stage liver disease; APRI, AST to platelet ratio index; FIB4, fibrosis index based on 4 factors; PALBI, platelet-albumin-bilirubin.

	AUC (95%CI)	P value
Patients with liver cirrhosis		
ALBI	0.63 (0.56−0.71)	<0.001
PALBI	0.60 (0.52−0.69)	0.031
MELD	0.55 (0.47−0.64)	0.069
APRI	0.56 (0.47−0.64)	0.289
FIB4	0.52 (0.43−0.61)	0.276
King's score	0.57 (0.48−0.66)	0.174
Patients without liver cirrhosis		
ALBI	0.62 (0.53−0.71)	0.012
PALBI	0.54 (0.46−0.62)	0.325
MELD	0.59 (0.51−0.67)	0.030
APRI	0.60 (0.52−0.68)	0.190
FIB4	0.61 (0.53−0.70)	0.011
King's score	0.63 (0.55−0.71)	0.007

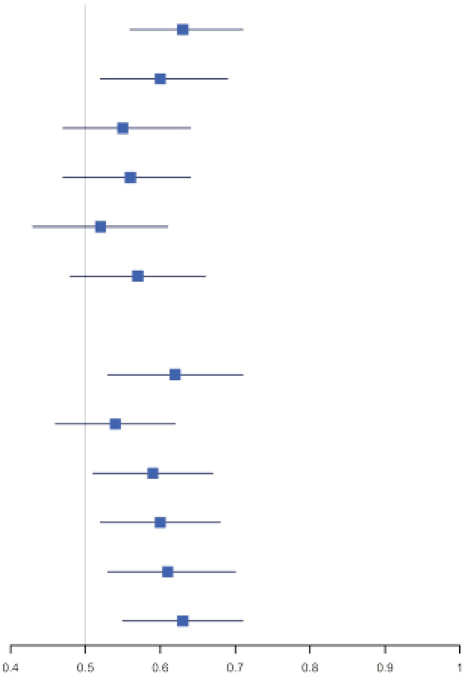

Figure S1.
